# Efficacy and safety of endovascular therapy for delayed hepatic artery post-pancreatectomy hemorrhage: development of extrahepatic collateral circulation and complications of post endovascular therapy

**DOI:** 10.1186/s42155-022-00326-x

**Published:** 2022-09-05

**Authors:** Yosuke Nozawa, Shinji Ymazoe, Koichi Masuda, Yutaka Takigawa, Yuko Kobashi, Koshi Ikeda, Takeshi Fukuda, Kenkichi Michimoto

**Affiliations:** 1 Department of Radiology, Tokyo Dental College Ichikawa General Hospital, 5-11-13 Sugano, Ichikawa-shi, Chiba, 272-8513 Japan; 2 Department of Radiology, Ushiku Aiwa General Hospital, 896 Shishiko-chou, Ushiku-shi, Ibaraki, 300-1296 Japan; 3 Department of Surgery, Tokyo Dental College Ichikawa General Hospital, 5-11-13 Sugano, Ichikawa-shi, Chiba, 272-8513 Japan; 4 Department of Radiology, Jikei University, Jikei University Hospital, 3-19-18 Nishishinbashi, Minato-ku, Tokyo, 105-8471 Japan

**Keywords:** Post-pancreatectomy hemorrhage, Transcatheter arterial embolization, Stent graft, Extrahepatic collateral circulation, Hepatic infarction

## Abstract

**Background:**

Endovascular therapy (ET) for delayed hepatic artery post-pancreatectomy hemorrhage (HA-PPH) may require complete hepatic artery occlusion (HAO). Nonetheless, the development of extrahepatic collateral circulation (EHC) and the relationship between radiological factors (EHC, portal vein stenosis, and HAO) and adverse hepatic events after ET remain unclear. This study aimed to evaluate the efficacy and safety of ET for delayed PPH and examine the development of EHC.

**Methods:**

A total of 19 ET cases for delayed HA-PPH were reviewed. Hepatic adverse events, portal vein stenosis, HAO, and mortality rate after ET were evaluated. Moreover, EHC from the left gastric artery (LGA), right inferior phrenic artery (RIPA), left inferior phrenic artery (LIPA), right internal thoracic artery (RITA), left internal thoracic artery (LITA), renal artery (RA), omental artery (OA), intercostal artery (IA), and branch of superior mesenteric artery (BSMA) was assessed using angiogram and computed tomography angiography (CTA).

**Results:**

All cases were successfully treated using transcatheter arterial embolization (*n =* 17) and stent-graft placement (*n =* 2) without mortality. EHC from the LGA (8/19), RIPA (10/19), LIPA (4/19), and RITA (3/19) was observed on post-ET CTA. The incidence of hepatic adverse events was significant in the group with both HAO and portal vein stenosis (*p <* 0.001) and absence of EHC from LIPA and RITA (*p <* 0.05).

**Conclusion:**

ET for delayed HA-PPH might be effective and safe. While avoiding both HAO and portal vein stenosis is important, the development of EHCs from LIPA and RITA may prevent hepatic adverse events after ET.

## Introduction

Delayed post-pancreatectomy hemorrhage (PPH) is relatively rare, with a prevalence of 1%–10%; however, delayed PPH is life-threatening, with a mortality rate of 11%–60% (Fang et al. 2018). The efficacy of endovascular therapy (ET) utilizing transcatheter arterial embolization (TAE) or stent-graft (SG) placement for delayed hepatic artery PPH (HA-PPH) is well-documented (Hwang et al. 2020; Shirai et al. 2021). SG placement effectively maintains hepatic artery patency; nevertheless, it is limited by the location of the bleeding point or arterial anatomical anomaly as well as the risk of acute obstruction (Shirai et al. 2021). Hepatic artery obstruction (HAO) is also often required in TAE for delayed HA-PPH.

Hepatic dysfunction and complications, including hepatic infarction and abscess after ET for delayed HA-PPH, are important clinical issues. Extrahepatic collateral circulation (EHC) that connects intrahepatic arteries and portal vein patency is associated with the prevention of hepatic complications after ET for delayed HA-PPH (Choi et al. 2021). Further, angiograms have mainly shown EHC from the left gastric artery (LGA) and right inferior phrenic artery (RIPA) (Choi et al. 2021; Sato et al. 2011). However, the possibility of other EHC resulting from HAO remains unelucidated. Accordingly, we aimed to evaluate the efficacy and safety of ET for delayed HA-PPH, and investigate the type and number of EHC on angiogram and computed tomography angiography (CTA) after ET.

## Materials and methods

This retrospective study was approved by our institutional review board (approval number: I 21–57), which waived the requirement for informed consent for the use of patient data.

### Patients

We reviewed 23 patients who underwent ET for delayed hemorrhage after pancreatic operations between October 1, 2013, and December 31, 2021, at our institution. Among them, 19 patients (15 men, 4 women; age: 69.84 ± 7.79 years) met our eligibility criteria, which comprised ET at the hepatic artery for hemorrhage occurring more than 24 h after pancreaticoduodenectomy or distal pancreatectomy. Four patients were excluded from this study according to the following exclusion criteria: splenic artery embolization for delayed PPH with CTA findings of pseudoaneurysm or extravasation (*n =* 3); not undergoing CTA before and after ET (*n =* 1).

ET for HA-PPH was decided based on CTA findings of pseudoaneurysm, extravasation, or irregular vessel shape with clinical hemodynamic instability.

### CT protocol

CTA images were obtained either using a 64-channel scanner (Brilliance, Phillips Healthcare, Cleveland, OH, USA) or a 320-channel scanner (Aquilion ONE, Canon Medical Systems, Otawara, Japan). The parameters for CT were as follows: 120 kVp; 250 mAs; rotation time, 0.5 s; pitch, 0.64 or 0.81; detector collimation, 64 × 0.62 or 80 × 0.50 mm; and scanning field of view (FOV), 350 or 320 mm.

The CTA protocol comprised non-contrast arterial and delayed phases of the whole abdomen (Table 1). Subsequently, an automatic power injector was used to administer a bolus of 600 mgI/kg of iodine contrast medium at a rate of 3.0–5.0 mL/s for 30 s. The contrast media included iohexol (iohexol 300 injection; Hikari Pharma, Tokyo, Japan) and iopamidol (iopamidol 370 or 300 injection; Fuji Pharma, Tokyo, Japan).

**Table 1:** CT angiography protocol

**1. Non-contrast phase**
**2. Arterial phase**
- An automatic power injector was used - A bolus of 600 mgI/kg of iodine contrast medium was administered at a rate of 3.0–5.0 mL/s for 30 s - The arterial phase was obtained at 10 s after reaching 150 Hounsfield units, with the region of interest being situated on the aorta, at the level of the celiac artery
**3. Delayed phase**
- The delayed phase was obtained at 90 s after administering the iodine contrast medium

All CTA images were obtained at end of inspiration. CTA axial and coronal images were reconstructed at a slice thickness of 1 and 2 mm for evaluation

*Abbreviations*: *CTA* Computed tomography angiography

The arterial phase was obtained at 10 s after reaching 150 Hounsfield units, with the region of interest being situated on the aorta, at the level of the celiac artery. Further, the delayed phase was obtained at 90 s after administering the iodine contrast medium. All CTA images were obtained at end of inspiration. CTA axial and coronal images were reconstructed at a slice thickness of 1 and 2 mm for evaluation. Subsequently, we analyzed the vessels using SYNAPSE VINCENT (Fujifilm, Tokyo, Japan). All 19 patients underwent presurgical, pre-ET (within 1 week before ET), and post-ET CTAs.

### Endovascular therapy

After local anesthesia, a 4- or 5-Fr sheath was inserted into the common femoral artery. Superior mesenteric artery (SMA) and celiac artery (CA) angiograms were obtained using 4- or 5-Fr angiographic catheters. The bleeding site and hepatic artery anatomy were confirmed using angiography. The pseudoaneurysm, parent artery of the pseudoaneurysm, or irregular vessel section related to the hemorrhage was tightly embolized using detachable coils (Target XXL, XL, soft, Stryker, Michigan, USA; Interlock, Boston Scientific, Massachusetts, USA; ED COIL, Kaneka Medical Products, Tokyo, Japan) and/or n-butyl-2-cyanoacrylate ([NBCA] Histoacryl, B. Braun, Hessen, Germany) using a microcatheter, at the operator’s discretion.

When using an SG, a 6-Fr or 7-Fr guiding sheath was inserted into the common femoral artery. Subsequently, an SG (VIABAHN; W. L. Gore & Associates, Delaware, USA) with a size of 6 mm × 2.5 cm or 7 mm × 2.5 cm was deployed at the parent artery of the pseudoaneurysm, followed by post-dilation with a balloon catheter.

### Evaluation

Technical success was defined by the disappearance of the pseudoaneurysm, extravasation, or irregular vessel shape on the post-ET angiogram. Discharge without arterial re-bleeding after ET was considered as a clinical success. Hemoglobin level, need for red blood cell transfusion, hospitalization period, and administration of intensive care unit were also assessed post-ET. Further, during the follow-up period after the discharge, late adverse events on CTA and the mortality rate were evaluated.

Hepatic dysfunction and radiological image findings after ET were evaluated, along with post-ET blood levels of aspartate aminotransferase (AST) and alanine aminotransferase (ALT). The maximum values of AST and ALT levels after ET were used for analysis. Criteria for Adverse Events version 5.0 and adverse events graded as ≥ 3 were regarded as significant (National cancer institute, 2022).

**Fig. 1:**
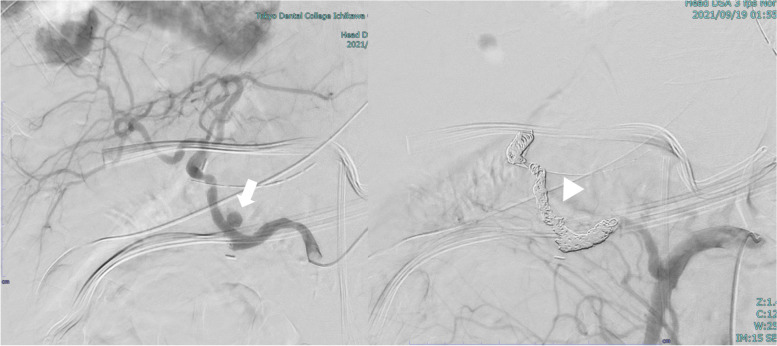
The common hepatic artery branched from the superior mesenteric artery; additionally, a pseudoaneurysm was observed in the proper hepatic artery (white arrow). Tight coil embolization was performed, which led to total occlusion of the native hepatic arterial flow (white arrowhead). Stent-graft placement was not performed due to the fact that the common hepatic artery branched with acute bending. In this case, a brachial approach might be effective if stent grafts are used

HAO was assessed on SMA and CA angiograms obtained immediately after ET. HAO was defined as the disappearance of arterial flow from the proper hepatic artery or both the right and left hepatic arteries (Fig. 1). Delayed HAO was also evaluated on CTA during the follow-up period.

**Fig. 2:**
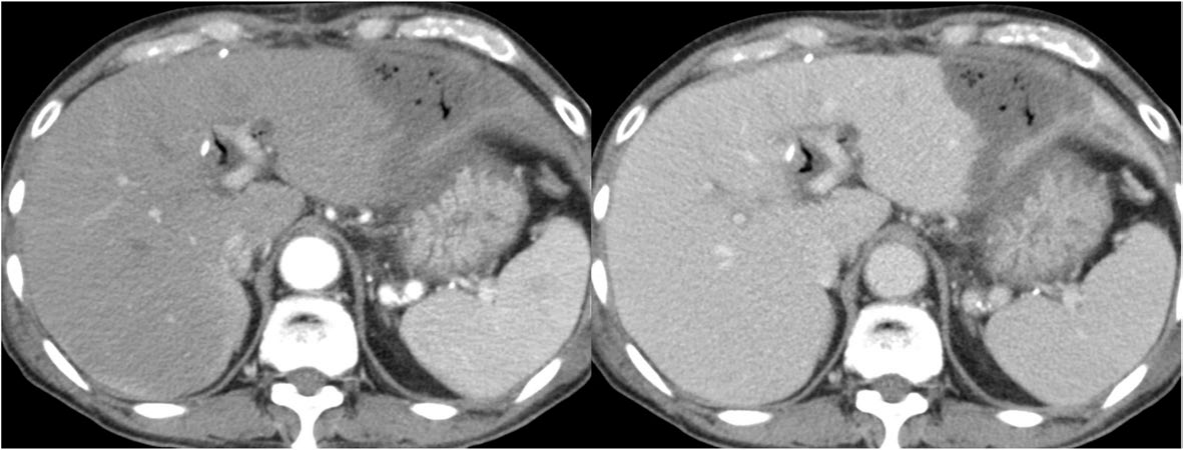
Hepatic infarction at segments 2 and 3 was observed in case 6 on the arterial and delayed phases of CTA after endovascular therapy

Portal vein stenosis > 50% on pre-ET CTA was considered as significant (Mine et al. 2014). Portal vein stenosis rate was defined as follows: portal vein stenosis rate = portal vein diameter on presurgical CTA- portal vein diameter on pre-ET CTA / portal vein diameter on presurgical CTA × 100. Hepatic infarction and abscess after ET were evaluated as adverse events during the same hospitalization period. Hepatic infarction was defined as an ill-defined wedge-shaped area of hypoattenuation on CTA without a mass effect on adjacent structures (Fig. 2). Hepatic abscess was also defined as a combination of presence of a hypoattenuation area with ring-enhancement on CTA and clinical symptoms of infection. Hepatic infarction and abscess were assessed according to the Cardiovascular and Interventional Radiological Society of Europe (CIRSE) classification system, and higher than grade 3 was considered significant (Filippiadis et al. 2017).

**Fig. 3:**
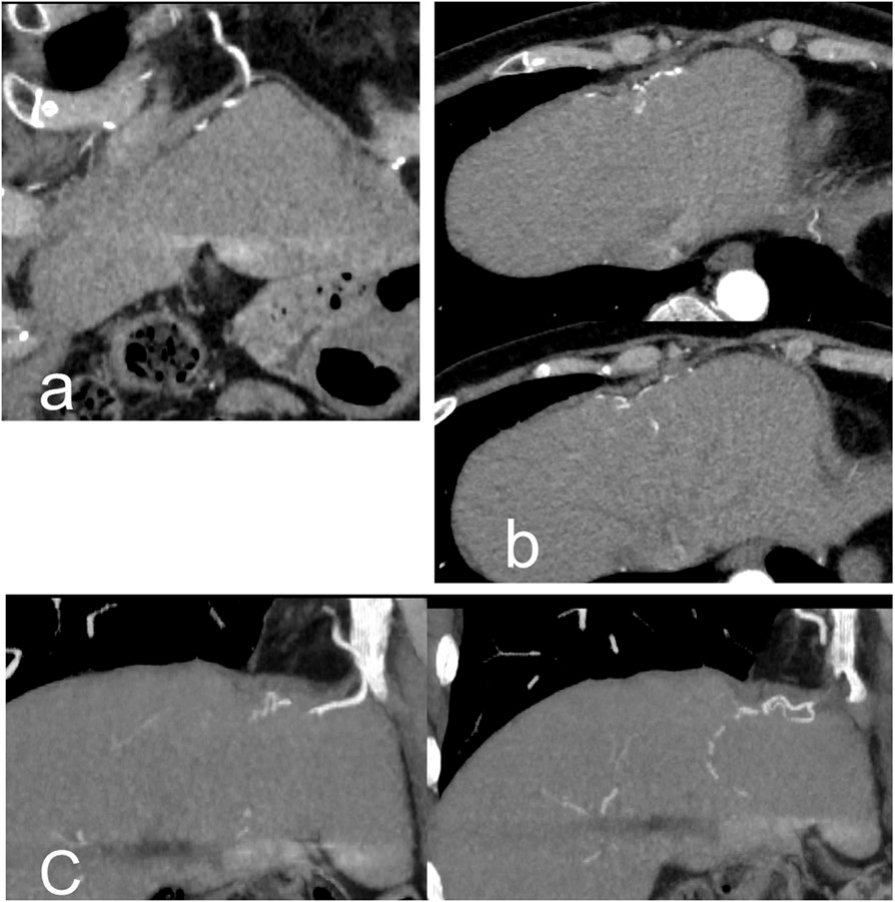
The right internal thoracic artery was connected to the intrahepatic artery through Sappey’s superior artery on the coronal (**a**), axial (**b**), and oblique (**c**) post-ET CTA images in case 5

Firstly, the possibility of an accessory hepatic artery and existing EHC that supply hepatic segments prior to ET was assessed on presurgical CTA images. Secondly, two interventional radiologists (Y.N. and K.M.) assessed EHC from the LGA, RIPA, left inferior phrenic artery (LIPA), right internal thoracic artery (RITA), left internal thoracic artery (LITA), renal artery (RA), omental artery (OA), intercostal artery (IA), and branch of superior mesenteric artery (BSMA) on angiographic and pre-ET and post-ET CTA images. EHC was defined by the appearance of a connection between these arteries and the intrahepatic arteries on abdominal angiography and/or post-ET CTA images, compared with presurgical CTA images (Fig. 3). Especially, EHC from LIPA, RITA, LITA, RA, OA, IA, and BSMA was defined as supplemental EHC.

### Statistical analysis

Continuous and categorical variables were analyzed using the Mann–Whitney U test and Fisher’s exact test, respectively. Statistical analyses were performed using the R software environment for statistical computing (www.r-project.org). Statistical significance was set at *p <* 0.05 (Kanda et al. 2013).

## Results

Table 2 summarizes the patient characteristics. The postoperative diagnoses included pancreatic carcinoma (*n =* 7), duodenal papillary carcinoma or adenoma (*n =* 3), common bile duct cancer (*n =* 3), intraductal papillary mucinous carcinoma (*n =* 2), endocrine tumor (*n =* 2), pancreatic metastasis of colon cancer (*n =* 1), and serous cyst neoplasm (*n =* 1). Further, the surgery operations included pancreaticoduodenectomy (*n =* 16) and distal pancreatectomy (*n =* 3). Hepatic function was assessed using the Child–Pugh score system (Child et al. 1964). The Child–Pugh scores before ET were A in 11 patients, B in 7 patients, and C in 1 patient. Hepatic artery anatomies were type 1 in 14 patients, type 3 in 2 patients, type 2 in 2 patients, and type 9 in 1 patient based on the Michels classification (Michels et al. 1966). Accessory hepatic artery and existing EHC that supply hepatic segments prior to ET were not observed on presurgical CTAs in any of the 19 cases.

**Table 2:** Patient characteristics

Characteristics	Patients (*n =* 19)
Age	69.84 ± 7.79
Sex (M:F)	16:3
Pancreatic fistula	19
Diagnosis	
Pancreatic cancer	**7**
Common bile duct cancer	3
Duodenal papillary carcinoma	2
Neuroendocrine tumor	2
Intraductal papillary mucinous carcinoma;	2
Duodenal papillary adenoma	1
Solid pseudopapillary neoplasm	1
Pancreatic metastasis	1
Child–pugh score	
A status	11
B status	7
C status	1
Michels classification	
Type 1	14
Type 2	2
Type 3	2
Type 9	1
Surgery	
Pancreaticoduodenectomy	16
Distal pancreatectomy	3
Clinical manifestation	
Sentinel bleeding	10
Gastrointestinal bleeding	6
Pseudoaneurysm incidentally detected on computed tomography scan	3

All 19 patients were diagnosed with pancreatic fistula, which was defined as drainage fluid amylase being more than three times the upper limit of the institutional standard at 3 days postoperatively (Pulvirenti et al. 2018). Sentinel bleeding signs before ET were observed in 10 (52.63%) patients; further, it was the most frequent clinical manifestation.

Table 3 shows the technical and clinical outcomes. All 19 ETs (100%) were successfully performed within 25.30 ± 25.78 days after pancreatectomy. SGs (6 mm × 2.5 cm and 7 mm × 2.5 cm) were used in cases 8 and 13, which maintained stent patency during the follow-up period. Only NBCA was used for pseudoaneurysm embolization in case 2, whereas coils alone or coils with NBCA were used in other cases. The most treated location was the region of gastroduodenal artery stump (57.89%). Before and after ET, hemoglobin level was not significantly changed (9.57 ± 1.73 and 9.47 ± 1.37 g/dl, respectively). Some (8 of 19) cases required red blood cell transfusion (5.75 ± 4.33 units) and 6 of 19 cases were admitted to the intensive care unit after ET. Only in case 10, SG treatment for portal vein hemorrhage was performed after initial ET. HAO was observed on angiogram after ET in 11 (57.89%) cases. EHC from the LGA (1/19), LIPA (2/19), and RITA (1/19) appeared on Pre-ET CTAs in cases 4, 5, 9, and 10 due to decrease of hepatic artery flow for PPH, and EHC from the LGA (8/19), RIPA (10/19), LIPA (4/19), and RITA (3/19) was observed on abdominal angiogram and/or post-ET CTAs. However, EHC from LITA, RA, OA, IA, and BSMA was not observed. Therefore, supplemental EHCs were recognized as valid in seven cases. The first post-ET CTAs were performed in 16.68 ± 16.14 days after ET.

**Table 3:** Technical and clinical outcomes

**Case**	**Surgery**	**Treatment site**	**Procedure**	**HAO**	**PV stenosis**	**AST /ALT elevation**	**Adverse Event**	**EHC**	**Post- ET CTA (day)**	**Additional Procedure**
1	DP	CHA	TAE with coils	No	No	No	No	LGA, LIPA	21	None
2	DP	PA of PHA	TAE with NBCA	No	No	No	No	RIPA	2	None
3	PD	RHA and PHA	TAE with coils	Yes	No	No	No	LGA, RIPA, LIPA	3	None
4	PD	GDA stump	TAE with coils	Yes	Yes	Yes	HA	LGA	2	None
5	PD	GDA stump	TAE with coils	Yes	No	No	No	RIPA, RITA	13	None
6	PD	GDA stump	TAE with coils	Yes	No	Yes	HA, HI	LGA, RIPA	3	None
7	PD	LHA	TAE with coils	No	Yes	No	No	None	14	None
8	PD	GDA stump	Stent-graft placement	No	No	No	HA	None	3	None
9	PD	GDA stump	TAE with coils and NBCA	Yes	No	No	No	RIPA. LIPA	6	None
10	PD	LHA and RHA	TAE with coils	Yes	No	No	No	RIPA. LIPA	19	PV Stent-graft placement
11	PD	GDA stump	TAE with coils	Yes	No	No	No	LGA, RIPA, RITA	43	None
12	PD	RHA	TAE with coils	No	No	No	HA	None	56	None
13	PD	GDA stump	Stent-graft placement	No	No	No	No	None	45	None
14	PD	GDA stump, RHA and MHA	TAE with coils	No	No	No	No	None	24	None
15	PD	GDA stump	TAE with coils	Yes	No	No	No	LGA, RITA	29	None
16	PD	GDA stump	TAE with coils and NBCA	Yes	Yes	Yes	HA	RIPA	9	None
17	PD	RHA and DPA	TAE with coils	No	No	No	No	None	19	None
18	PD	GDA stump	TAE with coils	Yes	Yes	Yes	HI	LGA, RIPA	4	None
19	DP	PHA, CHA and GDA	TAE with coils	Yes	Yes	Yes	HI	LGA, RIPA	11	None

*Abbreviations*: *ALT* Alanine aminotransferase, *AST* Aspartate aminotransferase, *CHA* Common hepatic artery, *DPA* Dorsal pancreatic artery, *EHC* Extrahepatic collateral circulation, *GDA* Gastroduodenal artery, *HA* Hepatic abscess, *HI* Hepatic infarction, *LGA* Left gastric artery, *LHA* Left hepatic artery, *LIPA* Left inferior phrenic artery, *MHA* Middle hepatic artery, *NBCA* n-butyl-2-cyanoacrylate, *PA* Pseudoaneurysm, *PV* Portal vein, *RITA* Right internal thoracic artery, *RIPA* Right inferior phrenic artery, *SA* Splenic artery, *TAE* Transcatheter arterial embolization, *TONHAF* Total occlusion of the native hepatic arterial flow

Significant portal vein stenosis was observed in five (26.31%) cases. The rate of portal vein stenosis on pre-ET CTA was 34.91 ± 21.85%. AST and ALT elevations after ET were observed in five cases. Hepatic infarctions after ET were observed in cases 6, 18, and 19. Case 19 could be conservatively treated without additional intervention; however, cases 6 and 18 required percutaneous drainage due to concomitant infection. Hepatic abscesses were observed in cases 4, 6, 8, 12, and 16. These five cases were treated with antibiotics. None of the patients developed mortality during hospitalization (49.94 ± 40.65 days) and they were all discharged without arterial re-bleeding.

Regarding the relation between clinical factors and adverse events (hepatic infarction and/or abscess) after ET (Table 4), univariate analyses demonstrated that both HAO and portal vein stenosis, AST and ALT elevation, and absence of supplemental EHC were statistically related to the adverse event rate after ET.

**Table 4:** Relation between clinical factors and adverse events after endovascular therapy

	***P*-value**
HAO	0.63
Both HAO and PV stenosis	** < 0.001**
Use of NBCA	1.00
Absence of supplemental EHC	**0.017**
Absence of EHC from LGA	0.37
Absence of EHC from RIPA	1.00
AST /ALT elevation (> CTCAE grade 3)	** < 0.001**

*Abbreviations*: *ALT* Alanine aminotransferase, *AST* Aspartate aminotransferase, *CTCAE* Common terminology criteria for adverse events, *EHC* Extrahepatic collateral circulation, *HAO* Hepatic artery obstruction, *LGA* Left gastric artery, *NBCA* n-butyl-2-cyanoacrylate, *PV* Portal vein, *RIPA* Right inferior phrenic artery

During the follow-up period, which was 704.0 days (range, 45–2120 days), none of the patients showed delayed HAO status and late hepatic adverse events. Five patients (26.31%; cases 1, 2, 3, 17, and 18) passed away due to progressive malignant disease; among them, four patients showed multiple hepatic metastasis.

## Discussion

We demonstrated the effectiveness and safety of ET for delayed HA-PPH and the development of EHC after ET. The technical and clinical success rates (100%) were comparable to those reported previously (Fang et al. 2018; Hwang et al. 2020; Cho et al. 2011). Although the need for red blood cell transfusion (42.1%) and admittance to the intensive care unit (31.5%) were observed at relatively higher rates in our study, arterial rebleeding was not observed in any of the cases, and this result was superior to 50.0% of previous reports. The hospitalization period was comparable with previous reports (Hwang et al. 2020; Farvacque et al. 2021).

SG treatment of ET can effectively maintain hepatic artery patency; however, there are several technical limitations (Hwang et al. 2020; Shirai et al. 2021). First, a larger sheath size is usually required in SG than in TAE. Second, it may be difficult to deliver the SG to the bleeding point in case of a steep tortuous and branching angle of the target artery. Moreover, even SG treatment involves the risk of occlusion leading to HAO status (Shirai et al. 2021; Gwon et al. 2011). While TAE has been used as the main treatment for delayed HA-PPH, it has been recognized as the procedure with more risk of HAO status depending on treatment site. In our study, adverse events (hepatic infarction and/or abscess) after ET were induced in all four cases with both HAO and portal vein stenosis (*p <* 0.001). Although we could treat hepatic infarction and abscess with only minimally invasive methods and observed no mortality in the same hospitalization, prophylactic portal vein stent placement may be acceptable in the case of more severe portal vein stenosis after ET for delayed PPH (Choi et al. 2021). Meanwhile, ET with only HAO might be acceptable in cases with sufficient patency of portal vein. In this study, we primarily used coils as an embolization agent (84.2%). NBCA was used in only 3 cases, and use of NBCA was not related to incidence of adverse events (*p* = 1.00). Embolization for pseudoaneurysm with NBCA in case 2 successfully prevented HAO status, but pseudoaneurysm embolization with NBCA has a high recurrent hemorrhage rate and risk for non-targeted embolization (Chang et al. 2019). While use of the combined methods of NBCA and coils in the 2 cases could be effective and safe for bleeding, they could not be treated with coils alone. NBCA could be trapped by the previously deployed coils without non-targeted embolization.

In addition, for preparation regarding these adverse events, AST and ALT elevation (> common terminology criteria for adverse events [CTCAE] grade 3) after ET might be a clinically useful predictor.

In our study, EHCs from LGA and RIPA (42.10% and 52.63%) were frequently observed, while the incidence rate of supplemental EHC from RITA (*n =* 3) and LIPA (*n =* 4) was 36.84%. Especially, the EHC from RITA after ET for delayed HA-PPH was the first to be demonstrated in our study. The RITA has anatomical potential for EHC and can be a feeder of hepatocellular carcinoma (HCC) (Choi et al. 2021; Moustafa et al. 2017; Nakai et al. 2001; Kim et al. 2007). Specifically, Hur et al. radiologically examined the EHC route from the RITA, which comprised the ensiform artery to Sappey’s superior artery (SSA) or the hepatic falciform artery (Hur et al. 2017). In our study, three patients showed EHC from the RITA that corresponded to the SSA route. Additionally, Kim et al. reported the EHC route from LIPA radiologically in 23 HCC cases, with the anteromedial limb route being the most frequent (Kim et al. 2009). We observed EHC from the LIPA through this route in four patients. Moreover, the absence of supplemental EHC is related to adverse event rate. Further, supplemental EHC tends not to manifest itself alone but is accompanied by the development of EHCs from LGA and RIPA in our study. Therefore, development of supplemental EHC might mean more enhancement of blood circulation to the liver after ET.

This study has some limitations, including its retrospective single-center design and the absence of randomization and comparison with SG. The main assessment of EHC was solely dependent on CTA. Further prospective, multi-institutional, randomized, large-scale studies are warranted.

In conclusion, ET with both HAO and portal vein stenosis status for delayed HA-PPH should be avoided, if possible, due to high risk of hepatic adverse events after ET; however, ET with HAO status might be acceptable in cases with sufficient patency of portal vein. Moreover, development of several EHCs was expected after ET. Especially, EHC from LIPA and RITA might prevent hepatic adverse events after ET for delayed HA-PPH.

## Data Availability

The datasets generated and/or analyzed during the current study are available from the corresponding author on reasonable request.

## Abbreviations

PPH: Post-pancreatectomy hemorrhage
ET: Endovascular therapy
TAE: Transcatheter arterial embolization
SG: Stent-graft
HA: Hepatic artery
HAO: Hepatic artery obstruction
EHC: Extrahepatic collateral circulation
LGA: Left gastric artery
RIPA: Right inferior phrenic artery
CTA: Computed tomography angiography
FOV: Field of view
SMA: Superior mesenteric artery
CA: Celiac artery
NBCA: N-butyl-2-cyanoacrylate
AST: Aspartate aminotransferase
ALT: Alanine aminotransferase
LIPA: Left inferior phrenic artery
RITA: Right internal thoracic artery
LITA: Left internal thoracic artery
RA: Renal artery
OA: Omental artery
IA: Intercostal artery
BSMA: Branch of superior mesenteric artery
HCC: Hepatocellular carcinoma
SSA: Sappey’s superior artery
CTCAE: Common terminology criteria for adverse events
CT: Computed tomography
PV: Portal vein
